# Selected advances in small-angle scattering and applications they serve in manufacturing, energy and climate change

**DOI:** 10.1107/S1600576723003898

**Published:** 2023-05-29

**Authors:** Andrew J. Allen

**Affiliations:** a Materials Measurement Science Division, National Institute of Standards and Technology, 100 Bureau Drive, Gaithersburg, MD 20899, USA; Brazilian Synchrotron Light Laboratory, Brazil

**Keywords:** small-angle X-ray scattering, small-angle neutron scattering, microstructure characterization, advanced manufacturing, energy applications, carbon dioxide reduction

## Abstract

Innovations in small-angle X-ray and neutron scattering (SAXS and SANS) at major X-ray and neutron facilities offer new characterization tools for researching materials phenomena relevant to advanced applications. For SAXS, the new generation of diffraction-limited storage rings, incorporating multi-bend achromat concepts, dramatically decrease electron beam emittance and significantly increase X-ray brilliance over previous third-generation sources. This results in intense X-ray incident beams that are more compact in the horizontal plane, allowing significantly improved spatial resolution, better time resolution, and a new era for coherent-beam SAXS methods such as X-ray photon correlation spectroscopy. Elsewhere, X-ray free-electron laser sources provide extremely bright, fully coherent, X-ray pulses of <100 fs and can support SAXS studies of material processes where entire SAXS data sets are collected in a single pulse train. Meanwhile, SANS at both steady-state reactor and pulsed spallation neutron sources has significantly evolved. Developments in neutron optics and multiple detector carriages now enable data collection in a few minutes for materials characterization over nanometre-to-micrometre scale ranges, opening up real-time studies of multi-scale materials phenomena. SANS at pulsed neutron sources is becoming more integrated with neutron diffraction methods for simultaneous structure characterization of complex materials. In this paper, selected developments are highlighted and some recent state-of-the-art studies discussed, relevant to hard matter applications in advanced manufacturing, energy and climate change.

## Abstract 2

Selected recent advances in small-angle X-ray and neutron scattering are highlighted, together with some of the hard material applications they serve in the areas of manufacturing, energy and climate change. This paper is associated with work presented at the 18th International Conference on Small-Angle Scattering, Campinas, Brazil, September 2022 (SAS 2022).

## 1. Introduction

In recent years, small-angle X-ray and neutron scattering (SAXS and SANS) methods have moved significantly beyond their traditional focus on the microstructure characterization of prepared samples, even though this will always be an important part of their remit. Small-angle scattering (SAS), encompassing SAXS and SANS, now plays an increasingly critical role in characterizing phenomena and processes within material systems that act over extended length scales (*e.g.* Krautwurst *et al.*, 2018 ▸; Djire *et al.*, 2019 ▸). This has been made possible by advances in detector technologies and beam optics, and in the ongoing development of optimized X-ray and neutron sources, resulting in faster counting times and measurements over more extended *q* ranges [where *q* = (4π/λ)sinθ, λ is the X-ray or neutron wavelength, and θ is one-half of the scattering angle]. These and other advances have enabled closely correlated measurements of SAXS with X-ray diffraction (XRD) (Li *et al.*, 2021 ▸) or SANS with neutron diffraction (ND) (Ioannidou *et al.*, 2021 ▸), or even total scattering measurements for short-range order (Kubota *et al.*, 2020 ▸). New instrumentation is also providing more integrated access to the lowest *q* values, regularly down to 1 × 10^−4^ Å^−1^ for ultra-small-angle X-ray scattering (USAXS) (Ilavsky *et al.*, 2018 ▸) or very small angle neutron scattering (VSANS) (Barker *et al.*, 2022 ▸), and down to 3 × 10^−5^ Å^−1^ for ultra-small-angle neutron scattering (USANS) (Barker *et al.*, 2005 ▸; Perry *et al.*, 2018 ▸). Other advances have been more specialized in nature, most notably, the widespread development and application of grazing-incidence SAXS and SANS (GISAXS and GISANS) (Timoshenko *et al.*, 2018 ▸) for studies of surface phenomena and comprehensive characterization of thin films and coatings. Two other developments should also be mentioned. In SAXS, X-ray photon-correlation spectroscopy (XPCS) or ‘speckle’ spectrometry, using small, coherent, X-ray beams, allows the internal dynamics of a wide range of material systems to be probed (Jo *et al.*, 2021 ▸; Chen *et al.*, 2021 ▸). Meanwhile, the development of polarized and spin-echo SANS capabilities (Schmitt *et al.*, 2020 ▸) has driven advances in studies of magnetic ordering, *e.g.* skyrmion dynamics (Kindervater *et al.*, 2019 ▸), as well as non-magnetic studies where spin echo can be a powerful probe (Gvaramia *et al.*, 2018 ▸; Biswas *et al.*, 2021 ▸).

One of the most important consequences of these new instrumental innovations has been a much greater integration of SAXS and SANS studies into overall materials research strategies. The ability to relate features and phenomena at the nanoscale contiguously with those at the micrometre scale (using USAXS, VSANS or USANS) or with corresponding structural phase information (using XRD or ND) without significantly disturbing the sample geometry or measurement configuration now plays a major role in a wide range of X-ray- or neutron-based materials research (Jia *et al.*, 2020 ▸). When combined with the faster counting times available in virtually all measurement configurations, these advances have opened up major opportunities for *in situ* or *operando* studies, conducted in real time under realistic sample environments (Wang *et al.*, 2020 ▸; Nigro *et al.*, 2021 ▸). The impact of new SAXS and SANS capabilities is well covered elsewhere for soft matter research (Motokawa *et al.*, 2019 ▸), for studies of magnetic ordering and magnetic alloys (Shu *et al.*, 2018 ▸; Mühlbauer *et al.*, 2019 ▸; Honecker *et al.*, 2022 ▸), and for geological research (Gadikota *et al.*, 2017 ▸; Vishal *et al.*, 2019 ▸). The potential for applications in these areas has clearly motivated some of the new SAXS and SANS instrumentation developments highlighted in following sections. However, the impact of new SAXS and SANS capabilities for other hard material applications is equally significant (dos Santos *et al.*, 2018 ▸). Hard matter applications include much of advanced manufacturing, especially for ceramics, alloys and their composites. They also encompass a broad range of realistic sample environments, especially attractive for *in situ* studies of complex materials phenomena relevant to advanced manufacturing processes, functional energy materials, and materials for climate change and carbon dioxide reduction. Thus, more emphasis is given here to hard matter applications.

In the sections that follow, selected recent advances in SAXS and SANS instrumentation are highlighted, but this is not intended as a comprehensive review of SAXS and SANS instruments around the world. Some examples will then be presented from the author’s own research collaborations, illustrating the relevance of recent SAXS and SANS developments for applications in manufacturing, energy and climate change. Finally, some future needs and prospects will be summarized.

## 2. Instrumental advances

Many new innovations in SAXS and SANS instrumentation have been realized over recent years or, being in the final stages of planning and design, will be offered soon. Here, some of these new innovations, either available now or planned to be available, are briefly highlighted with respect to specific facilities.

### 2.1. New instrument developments in SAXS

Major X-ray facility developments are increasingly trending towards multi-bend achromat (MBA) upgrades of synchrotrons to make them into diffraction-limited storage rings, as well as the construction of new X-ray free-electron laser (XFEL) facilities. These more coherent X-ray sources (fully coherent for XFELs) may not always be an advantage for SAXS if they oblige an XPCS approach for both measurements and analysis, which is not necessarily the desired condition for workhorse SAXS characterization. For most SAXS applications at diffraction-limited storage ring synchrotrons, the incident beam can still be made sufficiently large for coherent effects to remain insignificant, and the main advance over previous third-generation facilities is an overall smaller (and correspondingly more intense) incident beam. When coherent effects are to be exploited, usually by slit-defined small incident beams (typically dimensions of tens of micrometres), new coherent imaging modes are becoming available, some of which can directly complement the information obtained in SAXS measurements (Li *et al.*, 2019 ▸), as well as XPCS. The following subsections highlight new SAXS instrumentation at major facilities including some of the new rapid combinations of SAXS with other X-ray measurement modes, especially wide-angle X-ray scattering (WAXS), mainly providing XRD. Finally, recent advances in laboratory-based SAXS instruments are of interest owing to their potential importance for industrial use (as well as biological and pharmaceutical research), and these are discussed briefly.

#### 2.1.1. MAX IV: CoSAXS with coherent SAXS imaging

**Figure 1:**
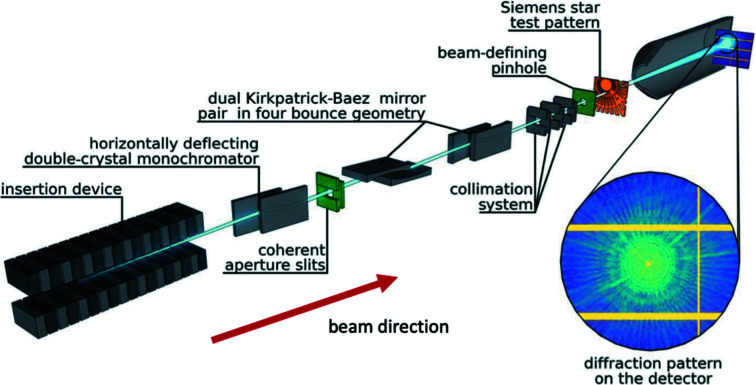
CoSAXS beamline [from Kahnt *et al.* (2021 ▸)]. From left to right: in-vacuum undulator, horizontally deflecting double-crystal monochromator (Si 111), coherence aperture slits (in green), two pairs of bendable KB mirrors, three slits minimizing parasitic contributions, a pinhole (in green) to limit the beam size, a Siemens star as sample (in orange) and a typical diffraction pattern on the hybrid pixel X-ray detector EIGER2 X 4M detector inside a vacuum vessel. (Note that the double-crystal monochromator remains in the vertical plane for most facilities elsewhere.)

The first major X-ray synchrotron facility to provide more partially coherent X-ray beams for SAXS based on the new state-of-the-art diffraction storage ring technology is the CoSAXS beamline (Plivelic *et al.*, 2019 ▸; Kahnt *et al.*, 2021 ▸) at the 3 GeV MAX IV synchrotron, Lund, Sweden. At this facility, the monochromator and (adjustable) beam-defining slits determine the longitudinal and transverse coherence of the incident X-ray beam, respectively. Fig. 1 ▸ 1 shows a schematic of the beamline, reproduced from Kahnt *et al.* (2021 ▸). Not all state-of-the-art SAXS facilities need all of the components shown here, but Fig. 1 ▸ is illustrative of X-ray beam components that can be considered for taking advantage of new synchrotron storage ring innovations. For CoSAXS, two pairs of vertical and horizontal focusing bendable Kirkpatrick–Baez (KB) mirrors are used to control the parallelism/divergence of the X-ray beam and define its focal position. The X-ray energy range is from 4 to 20 keV. Design predictions have indicated ≈10% coherence is possible for ≈7 keV X-rays with a total X-ray flux between 10^12^ and 10^13^ photons per second. Experimental verification using scanning X-ray ptychography has confirmed that viable XPCS measurements can be made at CoSAXS and can be combined with regular SAXS (or X-ray coherent imaging) without disturbing the sample configuration. While early applications are predominantly in the soft matter physics, chemistry and biology areas, colloidal particle dynamics, biomedical and other energy applications can clearly be accommodated. MAX IV is also commissioning another general-purpose SAXS instrument, ForMAX, which will combine SAXS with WAXS and XRD, as well as full-field tomographic imaging. With an available X-ray energy range of 5–25 keV and the ability to move easily between measurement configurations without disturbing the sample, this facility promises to have even more impact for hard materials applications (see https://www.maxiv.lu.se/beamlines-accelerators/beamlines/formax/).

#### 2.1.2. European Synchrotron Research Facility (ESRF): ID02 and ID31

**Figure 2:**
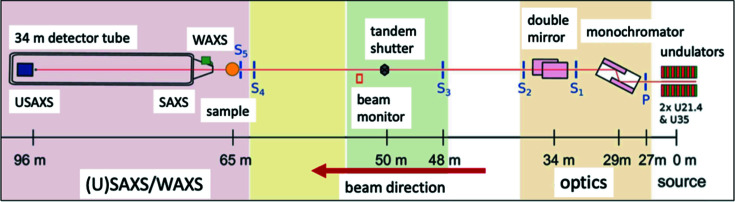
The schematic layout of the TRUSAXS (ID02) beamline [from Narayanan *et al.* (2022 ▸)], indicating the main components. Colors represent different sections of the beamline.

With the recent Extremely Bright Source (EBS) upgrade, beamline ID02 at the ESRF, working in an X-ray energy range of 8–20 keV and with a typical *q* range from 0.0001 to 5 Å^−1^, has become an even more versatile facility for *in situ* USAXS, SAXS and WAXS measurements with fast (sub-millisecond) time resolution. ID02 now incorporates rapid XPCS (speckle) and coherent imaging measurements for *q* < 0.01 Å^−1^ for studying transient processes without disturbing the sample configuration (Narayanan *et al.*, 2022 ▸). While the focus of ID02 has been traditionally in the soft matter physics area (Narayanan *et al.*, 2018 ▸), the ability to combine USAXS/SAXS with XRD, XPCS and coherent imaging has many advantages for research across the whole of materials science. Fig. 2 ▸ presents a schematic of the beamline, reproduced from Narayanan *et al.* (2022 ▸). The basic arrangement in the experimental hutch of the three main measurement configurations is indicated.

The experimental arrangement for ID02 essentially presents the template for several state-of-the-art synchrotron-based SAXS instrument designs. SAXS, including USAXS in the ultra-low *q* regime, is accomplished by positioning the high-resolution 2D detector in the detector tube to give the required sample-to-detector distance. Meanwhile, the WAXS detector for XRD and possible other imaging detector configurations are placed before the SAXS detector tube. XPCS can be achieved by using suitably small beam-defining slits. Rapid coverage of the large *q* range, as well as combined SAXS/XRD, is achieved by placement of large state-of-the-art 2D detectors requiring only short exposure times. As well as the potential for studies using ID02 across all areas of materials science, the EBS-upgraded ESRF also offers the high-energy beamline ID31 (X-ray energies 21–150 keV) for studies of hard materials, especially of buried interface structures and materials processing under a range of available sample environments. The ID31 portfolio includes SAXS, XRD, GISAXS and pair distribution function (PDF) analysis, providing further opportunities for SAXS research across much of materials science.

#### 2.1.3. Advanced Photon Source (APS): USAXS

**Figure 3:**
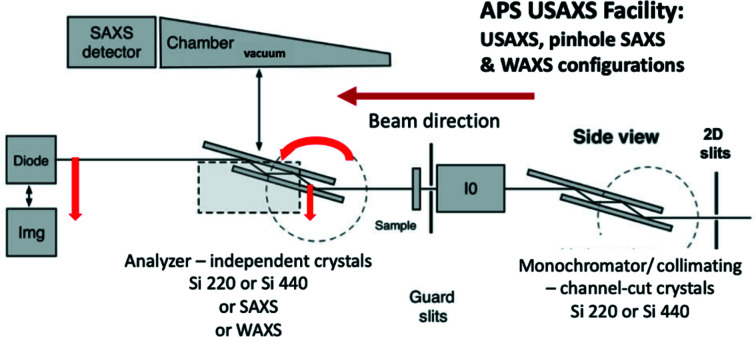
Schematic of the APS USAXS facility showing the interchangeable X-ray imaging camera, USAXS (photodiode and analyzer crystals), SAXS (detector with evacuated chamber) and WAXS (detector in air) instrument configurations. Coordinated motions for USAXS scanning shown in red. [I0 = ion chamber (beam monitor), Diode = USAXS detector, Img = X-ray imaging camera] – adapted from Ilavsky *et al.* (2018 ▸).

Several beamlines at APS incorporate SAXS and GISAXS capabilities, including high-energy SAXS (with X-ray energy up to 150 keV) at APS 1-ID and industrially focused SAXS at 5-ID (DND-CAT). However, the main USAXS/SAXS/WAXS workhorse for hard materials research remains the USAXS facility, recently located at APS 9-ID (Ilavsky *et al.*, 2018 ▸) but planned to become part of the APS 12-ID facility from mid-2024 following the APS MBA upgrade (APS-U). The APS USAXS facility uses Bonse–Hart crystal optics to provide the incident beam for all measurement modes and analyzer crystal optics for the core absolute-calibrated USAXS data collection itself. The USAXS photodiode detector and analyzer crystal assembly are interchanged with compact Dectris Pilatus 2D position-sensitive detectors for the SAXS and WAXS modes. The overall *q* range for USAXS/SAXS is 0.0001–1.5 Å^−1^ with overlapping WAXS (XRD) out to *q* ≈ 6.5 Å^−1^. However, use of higher X-ray energies up to 30 keV at 12-ID and higher-order crystal optics are projected to reduce the minimum *q* to 3 × 10^−5^ Å^−1^, due to a narrower crystal rocking curve Darwin width for higher-order diffraction optics. Fig. 3 ▸ presents a schematic of the measurement setup [adapted from Ilavsky *et al.* (2018 ▸)], as used at APS 9-ID. Coupled with appropriate pre-amplifier electronics, the photodiode detector (Diode) has an intensity linear dynamic range > 11 decades. This is key to the role of USAXS as a primary intensity calibration standard. Recently, the USAXS instrument stages have been replaced with a system that scans in the horizontal plane, anticipating the upcoming APS upgrade. The much greater stability afforded by vertical crystal rotation axes enables use of higher-order crystal reflections with narrower rocking curves and hence lower minimum attainable *q* values.

Note that, owing to dynamical scattering considerations, use of Si 220 or 440 (or many other *hkl*) X-ray optics while employing an Si(111) upstream monochromator results in the effective elimination of X-ray harmonics without the need for harmonic rejection mirrors (Zhang *et al.*, 2018*a* ▸). The new USAXS instrument stages operate significantly faster than the previous instrumentation, including the core USAXS flyscan operation. While USAXS flyscan time resolution cannot match the millisecond resolution possible with conventional SAXS detectors, after the APS upgrade, complete USAXS/SAXS/WAXS data should be obtainable within 2 to 3 min. Finally, it should be noted that the *q* resolution, *Δq*, is generally given by *q*_min_ for both USAXS and USANS Bonse–Hart instruments This is not necessarily the case for conventional SAXS and SANS instruments – even when an extended sample-to-detector distance and/or focusing optics put *q*_min_ into the Bonse–Hart range.

#### 2.1.4. Other synchrotron-based SAXS facilities

In addition to the major fourth-generation MBA upgrades, ESRF-EBS and APS-U, PETRA-IV in Germany presents a similar upgrade, and SPring-8 in Japan is also in the planning stages for an MBA upgrade. All of these facilities are, or will become, 6 GeV electron storage rings. They will be joined by a new fourth-generation synchrotron (also a 6 GeV storage ring), HEPS, under construction near Beijing, China. Following on from MAX IV in Sweden, other new facilities with electron storage energies in the 2–3 GeV range are also becoming available: *e.g.* SIRIUS, in Brazil, where the CATERETÊ coherent SAXS beamline (https://lnls.cnpem.br/facilities/caterete-en/) will offer coherent SAXS imaging capabilities building on instruments such as the present cSAXS (https://www.psi.ch/en/sls/csaxs) at SLS in Switzerland. Many other facilities are being upgraded (or upgrades are planned), *e.g.* Advanced Light Source (ALS-U) in the USA, ELETTRA-2 in Italy, SLS-II in Switzerland, SOLEIL-U in France and DIAMOND II in the UK. Many of these developments have been summarized recently by Shin (2021 ▸). While SAXS instruments feature at virtually all these facilities, the I22 SAXS/WAXS beamline at the Diamond Light Source in the UK represents a state-of-the-art workhorse user instrument (Smith *et al.*, 2021 ▸). The arrangement of the beamline components shares several of the features present at the MAX IV CoSAXS beamline or at ESRF ID02 (even before the DIAMOND II upgrade). In principle, the X-ray energy range is from 3.7 to 22 keV but 14 to 20 keV for microfocus SAXS applications. A typical SAXS *q* range is 0.001–0.3 Å^−1^ with overlapping WAXS to ≈6 Å^−1^. Millisecond time resolution can be approached in some experiments. Although originally emphasizing soft matter science, the I22 applications have broadened to include much of materials science, greatly helped by the availability of a wide range of sample environments.

#### 2.1.5. X-ray free-electron laser facilities: EuroXFEL–MID and LCLS–XCS

XFEL facilities are based on superconducting linac technologies. They can operate at MHz repetition rates such as are already offered at the European XFEL (EuroXFEL), Hamburg, Germany, or shortly will be at the Linac Coherent Light Source (LCLS), Stanford, CA, USA, with LCLS II. These facilities deliver pulsed, transversely coherent, hard X-rays with unprecedented flux and short pulse durations. Until recently, these next-generation X-ray facilities have not been associated with SAXS, given the destructive nature of the beam and the frequent need to reconstruct the sample structure from coherent data obtained often from a single pulse prior to destruction of the sample. However, some instruments at XFELs provide SAXS-relevant information of likely increasing interest in the future. Two instruments should be mentioned here: the Materials Imaging and Dynamics (MID) instrument at EuroXFEL (Madsen *et al.*, 2021 ▸) and the X-ray Correlation Spectroscopy (XCS) instrument at LCLS (Alonso-Mori *et al.*, 2015 ▸). In both cases, different detector segments can be used to collect coherent SAXS (including multi-speckle XPCS), WAXS (coherent XRD patterns) and imaging data. The high XFEL pulse repetition rate, together with the pulse-train structure not discussed here, provide major opportunities for microstructural and structural studies of rapid dynamics in materials through the use of methods such as MHz XPCS.

#### 2.1.6. New developments in laboratory-based SAXS

Laboratory-based SAXS instruments provide a workhorse for more routine measurements, including those in an industrial context, provide a test bed for experimental studies to be developed further at one of the major X-ray facilities, and can also provide an important teaching and training tool. Several laboratory SAXS instruments are now available commercially, from companies such as Xenocs (Grenoble, France), Anton Paar (Graz, Austria), Malvern Panalytical (Worcester, UK), Bruker AXS (Billerica, MA, USA), Rigaku (Tokyo, Japan) *etc*. Many of these designs are well optimized for calibration setup and optimized signal-to-noise ratio *etc*., and some suppliers also offer a compact Bonse–Hart USAXS design. X-ray sources with higher X-ray energies than Cu *K*a are now increasingly available, including instruments incorporating Excillum (Kista, Sweden) metal liquid sources, some of which can operate at X-ray energies > 24 keV. Elsewhere, laboratory-based SAXS instrument designs have been adapted and optimized for specialized use in the laboratory (Lyngsø *et al.*, 2021 ▸) or for users to extend synchrotron-based SAXS facilities temporarily (Pauw *et al.*, 2021 ▸).

### 2.2. New instrument developments in SANS

New innovations in SANS instrumentation combine the development and realization of new neutron facilities (mainly, but not exclusively, pulsed neutron sources), new neutron optics (lenses, prisms *etc*.), new detector technologies and new multi-detector configurations. While the incident neutron flux cannot match those at the major X-ray facilities, SANS counting times and measurement *q* ranges have been transformed by these developments in recent years. As with SAXS, long-detector-geometry SANS instruments are well established for providing access to low *q* values and overlap with Bonse–Hart USANS instruments that permit the lowest *q* values. This is a more critical need for USANS/SANS than for USAXS/SAXS because of the generally low counting rates for Bonse–Hart USANS instruments, despite major progress over the years (Strunz *et al.*, 1997 ▸; Barker *et al.*, 2005 ▸; Agamalian & Koizumi, 2011 ▸).

For long-detector-geometry SANS instruments, it is no longer always necessary to use very long wavelength neutrons (>18 Å) to access low *q*, which can have the disadvantage of the slow neutrons ‘falling’ under gravity over a long sample-to-detector distance and can also give rise to extensive multiple scattering in the sample. Low *q* can be achieved with modest neutron wavelengths (typically, 5–8 Å) by using neutron lenses or prisms to maintain a small and clean neutron beam geometry along the whole length of the SANS instrument, with a correspondingly small beam stop in front of the detector. The KWS-3 instrument at the Heinz Maier-Leibnitz Zentrum (MLZ) in Garching, Germany (Pipich & Fu, 2015 ▸), even incorporates an adjustable double-focusing toroidal mirror design, allowing the instrument to bridge the gap between USANS and SANS *q* ranges and support a wide range of technological materials research. In the subsections that follow, new innovations are highlighted at some of the major neutron facilities, both at reactor-based facilities where SANS is sometimes coupled with various imaging methods and at pulsed neutron sources where SANS is increasingly combined with wide-angle neutron scattering (WANS) for ND and/or total scattering PDF measurements. Polarized and spin-echo SANS are becoming more prominent at both types of facility but are not discussed in detail here. The faster overall counting times for obtaining SANS data over the whole *q* range now permit SANS studies to be much more integrated with other diagnostic methods under *in situ* or *operando* conditions.

#### 2.2.1. Institut Laue Langevin (ILL): D33

**Figure 4:**
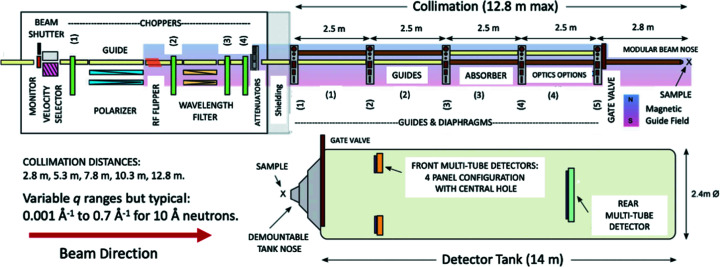
Schematic of ILL’s D33. The maximum collimation distance of 12.8 m and system of inter-collimation section diaphragms define the incident beam collimation. The 14 m long, 2.4 m diameter detector tank houses two movable detector trolleys accommodating five panels of multi-detectors [from Dewhurst *et al.* (2016 ▸)].

Long the world’s premier facility for cold neutron science, the ILL, Grenoble, France, has pioneered the concept of the long-flight-path SANS instrument with an optimized neutron cold source. It has delivered over the years D11 (lowest *q* for a conventional SANS instrument), D22 (highest flux) and, in the past decade, D33, ushering in a new era of highly versatile SANS instruments (Dewhurst *et al.*, 2016 ▸). Fig. 4 ▸ presents a schematic of the D33 setup.

D33 is flexible in terms of resolution and dynamic *q* range, offers both monochromatic and time-of-flight operation, and can accommodate a wide range of bulky sample environments and *in situ* processing. The large multi-tube detectors with their independent segments allow SANS data collection over much of the *q* range in a single measurement. This and the high neutron flux make D33 suitable for real-time measurements, even on weakly scattering samples. The instrument also offers a pulsed time-of-flight (TOF) mode (unusual for SANS at a reactor neutron source) with the advantage of an adjustable pulse frequency, which allows access to an enhanced dynamic *q* range, significantly beyond that attainable in monochromatic operation, and the opportunity for customized rapid real-time experiments. Beam polarization and polarization analysis options enable ambitious studies of advanced magnetic ordering. Various non-standard options are also possible, including multi-beams for low *q* and/or small samples, as well as scanning neutron imaging capabilities – with the ability to resolve both transmission and scattering contrast mechanisms.

#### 2.2.2. NIST Center for Neutron Research (NCNR): VSANS

**Figure 5:**
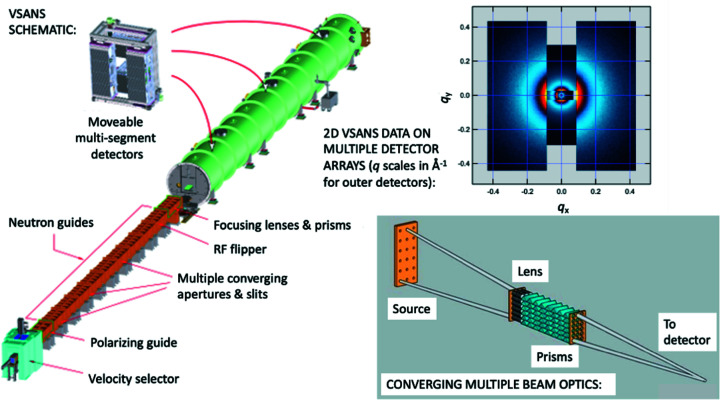
VSANS schematic (https://www.nist.gov/ncnr/chrns-vsans-very-small-angle-neutron-scattering), converging multiple beam optics [from Barker *et al.* (2022 ▸)] and typical 2D VSANS data on multiple detector segments (author’s data).

The VSANS instrument at NCNR, NIST, Gaithersburg, USA (Barker *et al.*, 2022 ▸) (see Fig. 5 ▸), builds on concepts introduced for D33 but also targets needs more specific to the NCNR user community, such as the need for greater overlap in *q* covered by ‘conventional’ SANS and the NCNR Bonse–Hart USANS instrument (Barker *et al.*, 2005 ▸).

The VSANS measurement range extends from *q* = 2 × 10^−4^ Å^−1^ to *q* = 0.7 Å^−1^, with data over much of the range collected simultaneously using three separate detector carriages on rails holding nine 2D detector arrays. Versatile collimation options and neutron wavelength selection allow the *q* resolution and beam intensity to be customized for specific experiments. This includes a highly oriented pyrolytic graphite monochromator option for studies requiring high-resolution analysis of small-angle diffraction peaks. High *q* resolution may also be achieved using multiple converging-beam collimation with circular pinholes, combined with refractive lenses and prisms. Relaxed vertical resolution with much higher beam intensity can be achieved with narrow slit collimation and a broad wavelength range using a Be crystalline filter and supermirror deflector to truncate the moderator source distribution below 4 Å and above 8 Å, respectively. Polarized beam measurements with full polarization analysis can be made using a high-performance supermirror polarizer and spin flipper, along with ^3^He polarization analysis. Like D33, the VSANS instrument is proving sufficiently versatile to support cutting-edge hard and soft matter research across the whole range of materials science. New onboard interferometric cold neutron imaging capabilities are also being developed for inhomogeneous materials that will complement the VSANS measurement scale range and time resolution.

#### 2.2.3. Australian Center for Neutron Scattering (ACNS): BILBY

The BILBY TOF SANS instrument at the Australian Nuclear Science and Technology Organisation (ANSTO) ACNS facility, Lucas Heights, NSW, Australia (Sokolova *et al.*, 2019 ▸), shares many of the innovations introduced with D33 at ILL and VSANS at NCNR. Like D33, BILBY includes a major TOF mode option, and like VSANS, BILBY is partly designed to increase the overlap at ACNS between conventional SANS using QUOKKA (Wood *et al.*, 2018 ▸) and Bonse–Hart USANS using KOOKABURRA (Rehm *et al.*, 2018 ▸). The possible overall *q* range is from ≈0.001 Å^−1^ to ≈1.8 Å^−1^. As well as enhancing the dynamic *q* range and enabling various rapid real-time measurements, the TOF option has been shown to improve the measurement resolution for small-angle diffraction peaks, as well as providing useful insights on inelastic scattering effects. As at ILL and NCNR, BILBY, together with the other ACNS SANS instruments, is opening up new SANS-based research across the whole remit of materials science, including hard material and geological research.

#### 2.2.4. Japan Proton Accelerator Research Complex (J-PARC): iMATERIA

**Figure 6:**
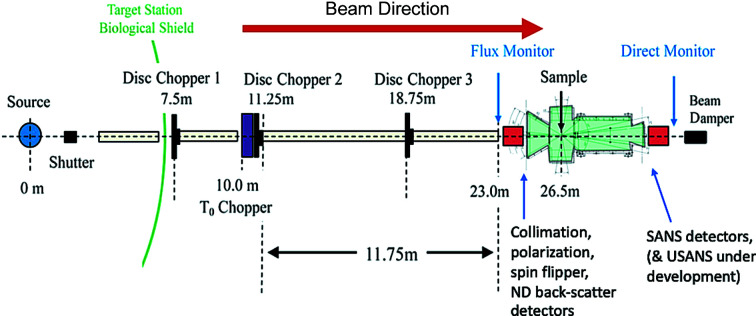
Schematic of iMATERIA TOF SANS at J-PARC [from Koizumi *et al.* (2020 ▸)].

Whereas recent SANS developments at reactor-based neutron sources have led to rapid data collection over much of the SANS *q* range in one detector configuration, together with a much more workable data overlap with the USANS *q* regime, SANS developments at pulsed neutron sources are making significant advances towards combining SANS and ND data collection using one measurement configuration – somewhat akin to the SAXS/WAXS capabilities that have matured in recent years at X-ray synchrotrons. One such state-of-the-art instrument currently operating is the TOF SANS instrument developed at the iMATERIA diffractometer, J-PARC pulsed neutron source, Tokai, Ibaraki, Japan (Koizumi *et al.*, 2020 ▸). Fig. 6 ▸ presents a schematic of the instrument, which features both forward- and back-scattering detectors for SANS and ND, respectively. The SANS *q* range is from 0.007 to 0.5 Å^−1^ while the ND range extends the maximum *q* to 30 Å^−1^. Complete SANS/ND data can be collected within a few minutes, and 1 min time resolution is possible for some measurements. The iMATERIA SANS instrument can also be used for grazing-incidence (GISANS or GI diffraction) studies and (as elsewhere at J-PARC) for polarized SANS magnetic studies or studies of hydrogeneous systems (Okudaira *et al.*, 2021 ▸; Noda *et al.*, 2020 ▸). USANS capabilities are also under development. This facility is enabling new research across all areas of materials science and is attracting significant industrial interest.

#### 2.2.5. Future evolution of SANS at pulsed neutron sources

The three most powerful pulsed neutron facilities currently are J-PARC in Japan, the Spallation Neutron Source (SNS) at Oak Ridge National Laboratory (ORNL), Oak Ridge, TN, USA (Heller *et al.*, 2018 ▸), and the ISIS facility on the Harwell campus, Dicot, UK. Several new facilities are planned or under construction around the world, but the most significant for SANS that will come online over the next decade are, first, the new European Spallation Source (ESS) under construction in Lund, Sweden, projected to be fully open in 2027, and then the Second Target Station (STS) project at SNS, expected to be completed in the early 2030s.

The STS will have a longer pulse repetition time than the first target station, allowing the use of longer-wavelength neutrons, and a major combined SANS/WANS instrument, CENTAUR, is planned (see https://neutrons.ornl.gov/centaur). CENTAUR will provide diffraction and spectroscopic capabilities for simultaneous time-resolved atomic- to meso-scale structure characterization in hierarchical systems under *in situ* or *operando* conditions. A contiguous *q* range from 0.001 to 20 Å^−1^ is envisaged, and the measurement time resolution is projected to approach seconds for many measurements. CENTAUR will also have polarization capabilities for magnetic and other studies.

Two complementary SANS instruments are planned for the initial ESS instrument suite: LoKI and SKADI (Jackson *et al.*, 2016 ▸; Andersen *et al.*, 2020 ▸; Jaksch *et al.*, 2021 ▸). LoKI will be a broad-band SANS instrument aimed primarily at soft matter applications with a maximum *q* range from 0.002 to 2 Å^−1^. Meanwhile, SKADI will be a versatile SANS instrument designed with high resolution and very low *q* SANS measurement capabilities, serving a broad range of materials science, and will have polarization capabilities for studies of magnetic ordering and hydrogeneous systems. The overall maximum *q* range will be from 0.0005 to 1 Å^−1^. Owing to the very high pulsed neutron flux at ESS, subsecond time resolution should be possible for many studies. Initial plans do not include simultaneous WANS (ND) capabilities, but these instruments should become the world’s most powerful SANS facilities. However, this will depend on the intensity dynamic range across the *q* range matching that at the best reactor-based SANS instruments.

### 2.3. Imaging modes and the upper size limit for SAXS and SANS

Various imaging modalities are emerging at major X-ray and neutron facilities to complement the microstructure information provided by SAXS and SANS. These include coherent diffraction imaging, ptychography, interferometric (grating) methods and various refraction imaging methods (Liebi *et al.*, 2015 ▸; Strobl *et al.*, 2016 ▸; Cao *et al.*, 2020 ▸; Withers *et al.*, 2021 ▸; Kahnt *et al.*, 2021 ▸). Imaging complements SAXS and SANS mainly by providing microstructure characterization at coarser length scales than SAXS and SANS can normally offer. Sometimes, coarse microstructures are the most readily related to changes in sample environment (pressure, temperature *etc*.) or to simultaneous property measurements (*e.g.* rheological, magnetic or electrochemical properties). Nevertheless, most imaging methods are being actively developed to improve their spatial resolution and achieve some scale overlap between imaging and SAXS/SANS microstructure characterization. For materials phenomena or processes occurring across many length scales, it is highly desirable to have some scale overlap in the features resolved by imaging and by SAXS/SANS, and to have the imaging measurement mode easily set up without disturbing the SAXS/SANS sample measurement configuration. Many of the SAXS and SANS instruments discussed above accommodate imaging options, but only a few have this automatically set up as an option during regular sample SAXS measurements.

Partly to increase the microstructure scale overlap with imaging, and in any case to maximize the dynamic range in length scales accessible to SAXS or SANS characterization, there is a continued interest in extending the USAXS or USANS *q* range to progressively smaller minimum *q* values, thus providing microstructure characterization at coarser length scales. However, USAXS/SAXS or USANS/SANS characterization of unknown microstructures is based on assuming the scattering to be in the Born approximation diffraction limit of ν ≤ 1, where ν is the phase difference between X-ray or neutron ‘waves’ passing through a scattering feature in the direction of *q* and those passing around it. For spherical scattering features with diameter 2*R* and an internal coherent scattering-length-density difference with the surrounding medium |Δρ| (where |Δρ|^2^ is the usual small-angle scattering contrast factor), and for X-rays or neutrons of wavelength λ, it can be shown (Berk & Hardman-Rhyne, 1988 ▸) that ν = 2*R*|Δρ|λ. Applying this criterion for spherical scattering features implies that a diameter, 2*R*, of a few micrometres gives ν ≈ 1 for typical SAXS or SANS. The situations for SAXS and SANS are broadly comparable because |Δρ| values are about an order of magnitude smaller for neutrons than for X-rays while typical λ values used for neutrons are about an order of magnitude larger than for X-rays. In practice the ν ≤ 1 criterion for the Born approximation is a soft limit and larger values of *v* up to ≈2 give rise only to subtle departures from the usual diffraction-based formulations used for interpreting SAXS and SANS data. For disordered scattering morphologies containing irregular arrangements of non-spherical features that encompass a range of length scales less than the coarsest dimension present, this criterion will be softened even further. Use of higher-energy X-rays (smaller λ) and the relatively small λ values of thermal neutrons used in Bonse–Hart USANS instruments (compared with cold neutrons for regular SANS) further mitigate the situation. Nevertheless, it is clear that there are ultimate limits to the maximum size of scattering features that can be characterized by SAXS or SANS before refraction effects cause size information for progressively larger features to be lost. Ultimately, for truly global microstructure characterization over all length scales, from sub-nanometre up to tens of micrometres and beyond, it will be important to accommodate imaging modalities among those readily set up during SAXS or SANS measurements in future instrument designs.

## 3. Selected current applications

Several potential applications of SAXS and SANS, or their variants, will benefit significantly from the recent and planned advances in instrumentation highlighted above, together with corresponding advances in experimental controls, sample environments, and data reduction and analysis. Many key areas of application have been the subjects of recent reviews. The following examples, based on application in recent years of current state-of-the-art SAXS and SANS instrumentation involving the author and collaborators, highlight hard materials research in support of advanced manufacturing, energy applications and climate change.

### 3.1. Delta-phase problem in additive manufactured Ni superalloy (SAXS)

**Figure 7:**
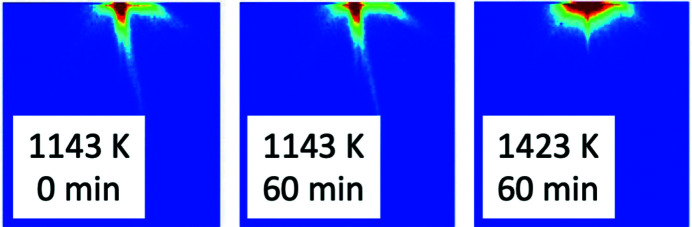
*In situ* SAXS streaking patterns for AM IN625 samples showing that streaks persist at the stress-relief temperature of 1143 K (870°C) but are removed at the homogenization temperature of 1423 K (1150°C) after 1 h [also see Zhang *et al.* (2017 ▸)]. Each image covers a *q*_*x*_, *q*_*y*_ range ≈ 0.6 Å^−1^, and the color scheme displays log intensities where red represents high intensity (around the beam stop) and blue low intensity.

Additive manufactured (AM) alloy components are effectively 3D-printed from digital computer-aided design (CAD) files. For Ni-based alloys, temperature transients during the AM build process can be as high as 10^6^ K s^−1^. This has the effect of causing local element segregation in the solid solution, with composition transients forming an Nb- or Mo-rich dendritic structure at the nanoscale level that can fall outside of the nominal alloy specification. In AM-built components composed of Ni superalloys such as IN625 (strengthened primarily by solid-solution hardening from Nb and Mo in an Ni–Cr matrix), such compositional spatial transients can lead to formation of potentially deleterious delta-phase (Ni_3_Nb) precipitates during the post-build stress-relief treatment essential for relieving high residual stresses arising during the AM build process. Fig. 7 ▸ shows streaks around the beam stop in 2D SAXS that can be attributed to the aligned dendritic structure in local composition (Zhang *et al.*, 2017 ▸). The length of the streaks in the SAXS data can be related quantitatively to the dendrite scale length. Fig. 7 ▸ indicates that the streaks persist during the post-build stress-relief heat treatment at 1143 K (870°C) but disappear within an hour during a subsequent homogenization treatment at a temperature of 1423 K (1150°C).

Used with complementary methods, including transmission electron microscopy, combined *in situ* USAXS/SAXS/WAXS measurements using 21 keV X-rays at APS have successfully identified, characterized and quantified the delta-phase formation process, including its activation energy, during post-build stress-relief treatments at temperatures up to 1143 K (870°C). These studies have confirmed that the subsequent homogenization heat treatment at 1423 K (1150°C) not only homogenizes the matrix, removing the dendrites, but also anneals out the delta-phase precipitates (Zhang *et al.*, 2018*b* ▸).

### 3.2. Electrochemical, phase and microstructure changes in SOFC electrodes (SAXS)

Research and development of solid-oxide fuel cell (SOFC) materials remains of current interest because of their growing role in carbon mitigation and reversible fuel reforming. Complex performance issues of the electrode materials and their resistance to degradation under the electrochemical environments and temperatures required for SOFC operation are a major focus. A promising family of potential new SOFC anode materials are the Sr_2_ *M*MoO_6−δ_ double perovskite series, where δ < 0.041 and *M* = Mg, V, Fe, Co or Ni have all been explored to varying extents (Witt *et al.*, 2020 ▸). In this study, a custom-built capillary cell was used to identify and quantify potential degradation reactions for the case of *M* = Co, denoted SCMO. *In situ* USAXS/SAXS/WAXS measurements (using 21 keV X-rays at the APS USAXS facility) were made with the sample under an appropriate reducing environment of flowing 4% mass H_2_/96% mass N_2_ gas. The temperature was ramped over several hours from 673 to 1098 K (400 to 825°C), covering the SOFC operational range, and the electrical conductivity across the sample continuously measured.

**Figure 8:**
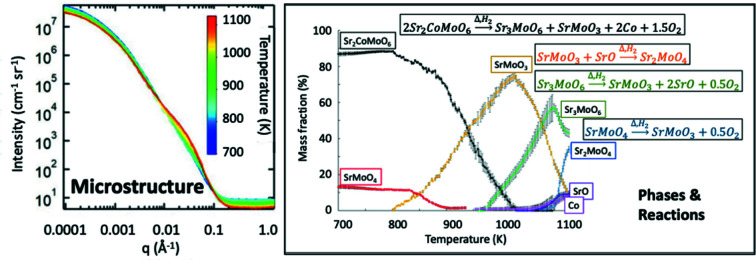
*In situ* slit-smeared USAXS/SAXS data for SCMO collected from 673 to 1098 K (400 to 825°C) under a flow of 4% mass H_2_/96% mass N_2_ gas, together with associated phase fraction variations plotted as a function of temperature for species formed during the reduction of SCMO. (Estimated standard uncertainties are smaller than the data traces for the USAXS/SAXS data and are given by the vertical bars for the composition variations.) Also shown in the right-hand plot are the expected reduction reactions for this system [in addition see Witt *et al.* (2020 ▸)].

Fig. 8 ▸ presents the time-dependent combined USAXS/SAXS data, clearly showing that microstructural changes occur during the temperature ramp. Using these data, together with corresponding WAXS data that provide the corresponding XRD patterns, it was possible to deduce the compositional variations as a function of temperature as shown in the right-hand plot of Fig. 8 ▸. Overall, the results indicated that, for the SOFC anode SCMO, continuous heating under a reducing atmosphere led to exsolution of Co from the double perovskite structure, formation of new strontium molybdate phases and a sharp rise in electrical conductivity. This occurred primarily between 825 K (≈ 550°C) and 1000 K (≈ 730°C), corresponding to the formation of the highly conductive SrMoO_3_ phase and finally Co itself. Clearly, combined USAXS/SAXS/WAXS studies such as this one can provide critical design information for future SOFC electrode materials.

### 3.3. Cold sintering of ceramics (SAXS)

**Figure 9:**
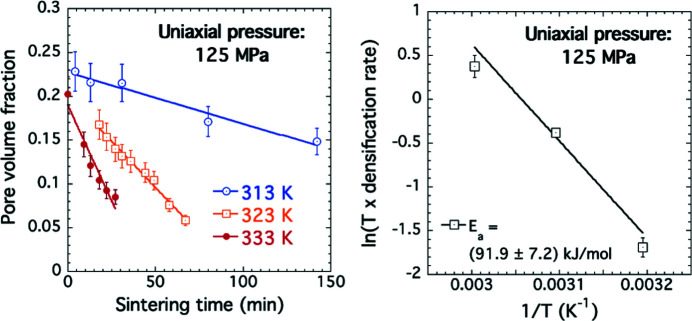
Arrhenius analysis of CSP of KDP for applied uniaxial pressure of 125 MPa, based on data for total pore volume fraction at 313 K (40°C), 323 K (50°C) and 333 K (60°C). The activation energy is for loss of porosity per mole of solid KDP filling the pore space during densification. Vertical bars are estimated standard uncertainties for modeled total porosities (left-hand plot) and computed standard uncertainties of the straight-line fit (right-hand plot). Also see Allen *et al.* (2022 ▸).

The cold sintering process (CSP), discovered by Randall and colleagues (Guo *et al.*, 2016 ▸), enables porous ceramic parts to be densified to over 95% full density at unusually low temperatures. Addition of water, an alkaline or acidic aqueous solution, or even a non-aqueous organic liquid can provide a transient solvent to facilitate densification (sintering) of a ceramic green body under uniaxial pressure (typically between 100 and 300 MPa) at a temperature between ambient and 600 K (≈ 300°C). At these markedly lower temperatures than required for conventional ceramic sintering (typically >1000°C), CSP offers reduced energy consumption and a reduced carbon footprint. However, many aspects of the process are not fully understood. Using a custom-built cold sintering stage, combined *in situ* USAXS, SAXS and WAXS (XRD) measurements at the APS USAXS facility, again with 21 keV X-rays, have enabled the sintering solid/pore morphology over nanometre to micrometre length scales to be followed and quantified during CSP while monitoring the presence of transient nanoscale, low-density, phases believed to develop at triple-point grain boundaries owing to solvent-mediated solid dissolution and reprecipitation at the grain/pore interface (Allen *et al.*, 2021 ▸, 2022 ▸). By conducting *in situ* CSP measurements at different temperatures, it has been possible to determine the activation energy for CSP, at least over a linear part of the densification range in a potassium di-phosphate (KDP) model system. The activation energy is indeed found to be 7 to 10 times smaller than that for conventional sintering (Fig. 9 ▸.)

These studies have subsequently moved on to CSP of a more technological functional ceramic: ZnO. However, much higher X-ray energies are needed to penetrate the densifying sample, requiring use of 70+ keV X-rays at the APS 1-ID high-energy X-ray beamline.

### 3.4. Additive manufacturing of cement (SANS)

Concrete incorporating Portland cement remains the primary construction material for infrastructure components, *e.g.* highways, bridges, dams, buildings. This has prompted a continued research focus on the hydration reactions between cement clinker and water. Degradation issues with concrete infrastructure, emerging new cementitious clinker materials, such as Portland–limestone cement (PLC) blends, and increasing interest in automated construction practices, such as 3D concrete printing (*i.e.* additive manufacturing), have placed new demands on the performance of cements. For 3D-printing applications, control of the time dependence of the hydration reactions can play a critical role in establishing a ‘printability’ window for extruded cement and concrete AM builds (Jones *et al.*, 2022 ▸). To investigate this issue, *in situ* dielectric rheo-SANS studies have been carried out at NCNR on hydrating cement pastes subjected to instrumented small-amplitude oscillatory shear, using an oscillatory (rotating drum) rheometer, to give the real and imaginary parts of the shear modulus and viscosity, as well as simultaneous measurements of the electrical conductivity across the cement sample, all as a function of cement hydration time for the first 12–24 h of cement hydration. The electrical and rheological properties of the hydrating paste can be related to fits of a long-standing fractal microstructure model, developed for interpreting SANS data from hydrating cement (Allen, 1991 ▸).

**Figure 10:**
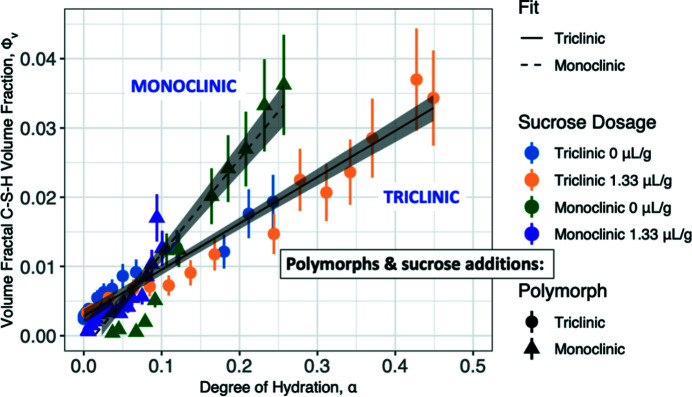
Volume-fractal C-S-H volume fraction, Φ_V_, versus degree of reaction (hydration), α, with straight-line fits. (Vertical bars show 95% confidence uncertainties.) Effects of sucrose addition collapse out but triclinic versus monoclinic distinction remains [also see Jones *et al.* (2022 ▸)].

The Jones *et al.* (2022 ▸) study shows that the decline in the loss tangent, *G*′′/*G*′ (where *G*′ and *G*′′ are the real and imaginary parts of the shear modulus, respectively), to its equilibrium ‘elastic’ value occurs at a hydration time that coincides with the onset of decline in the electrical conductivity, σ (due to loss of water-filled pore percolation across the sample), and the onset of significant calcium–silicate–hydrate (C-S-H) gel developing in a volume-fractal structure between the clinker grains to bind the cement grains together. Furthermore, when applied to the hydration of the two purified polymorphs (monoclinic and triclinic) of tricalcium silicate (denoted C_3_S), the most prominent active ingredient of Portland cements, this study has shown that, while the two polymorphs result in different developing hydrated morphologies, addition of sucrose to retard the hydration reactions does not affect the respective morphologies for a given degree of reaction, α, determined from calorimetry (see Fig. 10 ▸). This suggests that the hydration time for a specific cement blend can be tuned for 3D printability without significantly perturbing the final morphology (and hence properties).

Current research is applying these results to rheo-SANS studies of more technologically important PLC systems for 3D printing, where the extended *q* range of the NCNR VSANS instrument allows the limestone particle size distribution to be incorporated seamlessly into multi-component fractal models of the hydrating cement microstructure.

### 3.5. CO_2_ adsorption in metal–organic framework sorbents (SAXS and SANS)

Research on carbon sorbent materials, such as metal–organic frameworks (MOFs), involving combinations of SAXS and SANS with small-angle diffraction and with regular XRD and ND is of increasing importance because of urgent priorities to address climate change issues and ultimately to bring about carbon dioxide reduction (CDR) in the atmosphere. MOFs also have potential roles in other selective gas adsorption applications including their use with supercritical CO_2_ as a solvent in catalysis applications. This situation is prompting advances in gas sorption sample environments at both SAXS and SANS facilities. Quantitative analysis of SAXS and SANS data can determine the degree to which local scattering contrast factors are modified by gas adsorption, and adsorption of gas into the flexible MOF cell structure causes changes in the peak positions and *d* spacings of some diffraction peaks, but not others. Combined SAXS/WAXS studies frequently enable the microstructural and structural aspects to be studied, rapidly and *in situ*, together, while SANS allows competing effects due to H_2_O (and D_2_O) adsorption to be quantified more sensitively.

**Figure 11:**
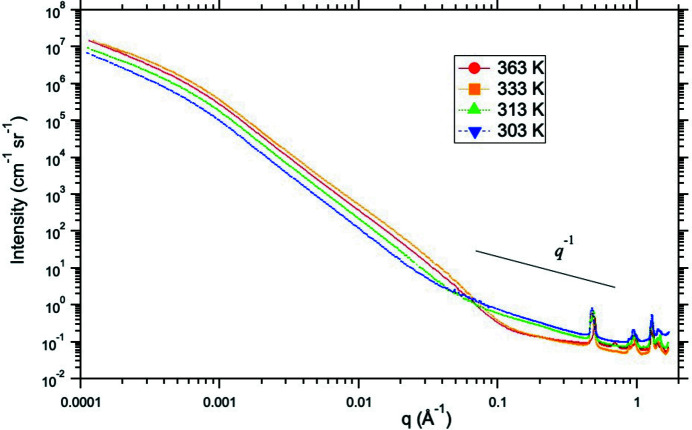
Combined, slit-smeared, USAXS/SAXS data for NiBpene PICNIC system during cooling from 363 K (90°C) to 303 K (30°C) under 76–80 bar CO_2_ pressure. Uncertainties are represented by the (small) scatter within each data set [also see Allen *et al.* (2019 ▸)].

In several studies of the ‘PICNIC’ family of MOF-like materials (Allen *et al.*, 2015 ▸, 1991 ▸, 2023 ▸), the microstructure and structure have been characterized by SAXS or SANS under single and dual gas conditions, as well as under high-pressure static CO_2_ conditions. Fig. 11 ▸ shows combined USAXS/SAXS data for the NiBpene PICNIC system, measured using a capillary gas system at the APS USAXS facility with 21 keV X-rays. The data displayed show the effects on the microstructure and structure of cooling the sample under supercritical CO_2_ gas pressure from the supercritical CO_2_ regime, into the liquid CO_2_ regime (303 K or 30°C data). The microstructural interpretation and structural (*d* spacing) changes in such studies (including inferred SAXS scattering contrast factor changes) can be compared with predictions of density functional theory to gain insights into the sorption behavior of such new and novel gas sorbent materials.

## 4. Concluding remarks and future directions

Given new developments in SAXS and SANS facilities, it is useful to summarize the evolving roles of SAXS and SANS research that can and will follow from these new capabilities. Global requirements for open data, open research and publication, as well as for independent validation of research results, are changing the environment for scientific research as a whole. Thus, it is also timely to indicate ways in which SAXS and SANS capabilities (and researchers) are moving to address these requirements.

### 4.1. Concluding remarks on current status of SAXS and SANS

In addition to their traditional role in microstructure characterization of stand-alone samples, SAXS and SANS are playing increasingly pivotal roles during *in situ* or *operando* research studies across all classes of materials systems and phenomena. This has been made possible both by development of more sophisticated sample environments and by effectively reduced measurement (counting) times. Inevitably, these developments present new high-throughput data reduction and analysis challenges, and virtually all major X-ray and neutron facilities are developing software and online tools to address these challenges. While data reduction is conducted using platforms and software tools developed at individual facilities, data analysis is made available to the whole SAXS and SANS community. Examples are *Irena* (Ilavsky & Jemian, 2009 ▸), *SASView* (https://www.sasview.org), the NIST SANS/USANS routines (Kline, 2006 ▸), *SAXS­utilities2* (Sztucki, 2021 ▸), and the SINQ instrument control system (*SICS*) (Heer *et al.*, 1997 ▸) which includes data acquisition and control.

Among instrument developments, SAXS capabilities at major facilities are combining SAXS with XRD in a single measurement, with fine time resolution (second or sub-second). Use of high-energy X-rays is connecting these capabilities to applications involving challenging sample environments and hard materials. Meanwhile, new SANS facilities continue to come online that allow data collection associated with nanometre-to-micrometre length scales in single measurement configurations with improved time resolution windows (minutes), also enabling more phenomena affecting hard materials to be probed under *in situ* conditions. Both SAXS and SANS are increasingly used with other diagnostic measurements, *e.g.* rheology, electrical impedance, diffraction and XAFS, as well as imaging, including coherent or interferometric imaging for both X-rays and neutrons. At pulsed neutron sources, SANS is becoming more coupled with neutron diffraction methods.

For SAXS, the field is still at the start of the MBA/diffraction-limit storage ring/XFEL revolution. Although not discussed in detail here, SANS with polarized neutrons and spin-echo capabilities will play an increasingly important role in key fields, such as the development of materials with advanced magnetic properties. Overall, the opportunities afforded by recent and emerging innovations affecting SAXS and SANS instrumentation are likely to have far-reaching consequences on the ways in which future SAXS- and SANS-based materials research will develop.

### 4.2. Future prospects

Coupled with the challenges of higher data rates and the need for rapid data reduction/analysis throughput, both for SAXS and SANS measurements themselves and for paired techniques such as XRD, ND *etc*., are growing requirements for open data and open publication (cOAlition S, 2020 ▸; UNESCO, 2021 ▸; International Science Council, 2021 ▸) and the need for consistent publication standards. To address these issues, there are growing international efforts, involving major X-ray and neutron facilities, to establish common data formats and standards for metadata (Könnecke *et al.*, 2015 ▸) that will satisfy FAIR data requirements (Wilkinson *et al.*, 2016 ▸), cross-platform reduction and analysis tools, and even research journal publication guidelines (Trewhella *et al.*, 2023 ▸). Ultimately, there is a need to establish research data requirements where all tasks – data collection and formatting with metadata for publication, data review and validation, *etc*. – are defined and achievable in both time and cost for future generations of researchers utilizing SAXS and SANS techniques. While this is a daunting task, a number of inter-related international efforts are underway, specific to SAXS and SANS research. Among these, the Small Angle Scattering Biological Data Bank, SASBDB (https://www.sasbdb.org/), encompasses a broad range of soft matter SAS-based research. Meanwhile, the canSAS collaboration (https://www.cansas.org/) includes the SAS Portal repository of openly available reduction and analysis software packages (http://smallangle.org/content/Software). These efforts will continue to strengthen the place of SAXS and SANS in materials research in general. At the same time, artificial intelligence (AI) and machine learning (ML) are becoming more integral to materials research. There are prospects at many major X-ray and neutron facilities to marry supercomputing AI/ML developments with incoming experimental facility data, both compatible with requirements set out above and enhancing further the role of SAXS and SANS in active ongoing research as well as future research paradigms based on data mining.

Finally, the development of new compact X-ray sources and, while not discussed here, development of compact neutron sources (see https://www.imoh.eu) will allow laboratory-based SAS facilities to play a stronger role in prototype studies, teaching and education, and industrial research. It will be important that the laboratory-based SAS research community is able to share fully in the developments at the major facilities highlighted in this paper.

## Footnotes

1 Certain commercial instruments, materials, software or processes are identified in this paper to adequately specify the experimental procedure. Such identification does not imply recommendation or endorsement by the National Institute of Standards and Technology, nor does it imply that the instruments, materials or processes identified are necessarily the best available for the purpose.
